# Prevalence of SARS-CoV-2 Infection Among Asymptomatic Health Care Workers in the Greater Houston, Texas, Area

**DOI:** 10.1001/jamanetworkopen.2020.16451

**Published:** 2020-07-27

**Authors:** Farhaan S. Vahidy, David W. Bernard, Marc L. Boom, Ashley L. Drews, Paul Christensen, Jeremy Finkelstein, Roberta L. Schwartz

**Affiliations:** 1 Center for Outcomes Research, Houston Methodist Research Institute, Houston, Texas; 2 Houston Methodist Neurological Institute, Houston Methodist Hospital, Houston, Texas; 3 Houston Methodist Academic Institute, Houston, Texas; 4 Department of Pathology and Genomic Medicine, Houston Methodist Hospital, Houston, Texas; 5 Weill Cornell Medical College, New York, New York

## Abstract

This cross-sectional study examines rates of severe acute respiratory syndrome coronavirus 2 (SARS-CoV-2) infection among asymptomatic health care workers and community residents in the greater Houston, Texas, area.

## Introduction

Asymptomatic severe acute respiratory syndrome coronavirus 2 (SARS-CoV-2) infection continues to be a major public health concern.^1^ Health care workers (HCWs) are at higher risk of infection and can become inadvertent vehicles of transmission.^2^ Therefore, Houston Methodist initiated a coronavirus disease 2019 (COVID-19) surveillance program among asymptomatic HCWs and expanded to asymptomatic community residents. We report prevalence of SARS-CoV-2 among the first group tested.

## Methods

This cross-sectional study was approved by the Houston Methodist institutional review board as part of a quality improvement project that includes a waiver of informed consent from HCWs, per institutional policy. Community residents were recruited via telephone, and written informed consent was obtained in person. This study is reported following the Strengthening the Reporting of Observational Studies in Epidemiology (STROBE) reporting guideline.

Houston Methodist comprises an academic medical center with 7 community hospitals treating patients with COVID-19. The HCWs included clinical employees in patient care areas, with and without patients with COVID-19, and nonclinical workers with no patient contact. Within COVID-19 units, certain job categories may have greater patient exposure, so we further classified COVID-19–facing HCWs into 5 job categories: (1) nursing (ie, registered nurses, nurse aides, bedside technicians, and emergency medical technicians), (2) clinicians (ie, physicians, residents, nurse practitioners, and physician assistants), (3) allied healthcare workers (ie, therapists, nonbedside technicians, pharmacists, and social workers), (4) support staff (ie, housekeeping and security), and (5) administrative or research staff (ie, managers, coordinators, administrative assistants, researchers, and research assistants).

From March 11 to April 19, 2020, we collected nasopharyngeal swabs, age, and sex information from self-reported asymptomatic HCWs and community residents. Testing was conducted via 1 of 3 cross-validated reverse transcriptase–polymerase chain reaction (RT-PCR) assays.

We report proportions with 95% CIs and used χ^2^ proportional trend test to explore the association between SARS-CoV-2 positivity and HCW subgroups. We also provide logistic regression-based sex and age adjusted odds ratios (aORs) for SARS-CoV-2 positivity across 7 hospitals and 5 job categories among COVID-19–facing HCWs. Analyses were performed using Stata statistical software version 16 (StataCorp). *P* values were 2-sided, and statistical significance was set at .05.

## Results

A total of 2872 individuals, including 2787 HCWs and 85 community residents, were included; the mean (SD) age was 40.9 (11.7) years and 73% (95% CI, 71.6%-74.9%) were women. In all, 3.9% (95% CI, 3.2%-4.7%) tested positive for SARS-CoV-2. Among clinical HCWs, 5.4% (95% CI, 4.5%-6.5%) from COVID-19 units and 0.6% (95% CI, 0.2%-1.7%) from non–COVID units had RT-PCR test results positive for SARS-CoV-2 (aOR, 9.10; 95% CI, 3.33-24.82). None of the nonclinical HCWs or community residents had positive test results (*P* for trend <.001) (Table 1).

**Table 1.** Age, Sex, and SARS-CoV-2 Positivity Proportions Among 3 Categories of Asymptomatic Health Care Workers and Community Residents

Characteristic	% (95% CI)				
	Overall sample (N = 2872)	Health care workers			Community residents (n = 85)
		Clinical		Nonclinical (n = 170)	
		COVID-19–facing (n = 1992)	Non-COVID-19–facing (n = 625)		
Age, mean (SD), y^a^	40.9 (11.7)	39.5 (11.3)	43.0 (12.1)	45.9 (12.3)	48.2 (9.8)
Women	73.3 (71.6-74.9)	74.6 (72.6-76.4)	72.5 (68.8-75.8)	54.7 (47.2-62.0)	87.1 (78.1-92.7)
SARS-CoV-2 positivity	3.9 (3.2-4.6)	5.4^b^ (4.5-6.5)	0.6^b^ (0.2-1.7)	0 (0 to <0.1)^c^	0 (0 to <0.1)^c^

Abbreviations: COVID-19, coronavirus disease 2019; SARS-CoV-2, severe acute respiratory syndrome coronavirus 2.

^a^ Missing: n = 10 (0.3%).

^b^ COVID-19–facing asymptomatic clinical health care workers were at a 9.1 times greater likelihood of testing positive for SARS-CoV-2 compared with non–COVID-19–facing clinical health care workers (adjusted odds ratio, 9.10; 95% CI, 3.33-24.82).

^c^ One-sided 97.5% binomial exact CI.

Among 1992 HCWs in units caring for patients with COVID-19, the rate of SARS-CoV-2 positivity ranged between 3.6% (95% CI, 1.3%-9.1%) for support staff to 6.5% (95% CI, 3.9%-10.7%) for allied health and 6.5% (95% CI, 3.6%-11.3%) for administrative staff. However, the proportions of participants with postive results for SARS-CoV-2 were not significantly different across the 5 job categories of COVID-19–facing HCWs (*P* for trend = .67).

After adjusting for age, sex, and job category, 2 hospitals demonstrated significantly higher likelihood of SARS-CoV-2 positivity among COVID-19–facing HCWs compared with the academic medical center (hospital 3: aOR, 2.78; 95% CI, 1.76-4.39; hospital 5: aOR, 2.49; 95% CI, 1.23-5.02), whereas the infection rate was significantly lower in another facility (hospital 2: aOR, 0.34; 95% CI, 0.12-0.95) (Table 2).

**Table 2.** Multivariable Model–Based Likelihood Estimates of SARS-CoV-2 Positive Results Across Different Hospitals and Job Categories for Asymptomatic Coronavirus Disease 2019–Facing Health Care Workers

Variable	Individuals, No.		SARS-CoV-2 Positivity, %	aOR (95% CI)^a^
	At risk	Tested		
Facility^b^				
1	1718	935	5.0	1 [Reference]
2	392	231	1.7	0.34 (0.12-0.95)
3	775	296	12.5	2.78 (1.76-4.39)
4	632	189	3.2	0.63 (0.27-1.51)
5	314	97	11.3	2.49 (1.23-5.02)
6	304	148	0	NA
7	554	96	3.1	0.62 (0.19-2.03)
Job category				
Nursing	2673	1281	5.3	1 [Reference]
Clinicians	562	210	5.7	1.09 (0.57-2.11)
Allied health	749	215	6.5	1.35 (0.73-2.51)
Support	398	112	3.6	0.80 (0.69-2.74)
Administration or research	307	170	6.5	1.38 (0.69-2.74)

Abbreviations: aOR, adjusted odds ratio; NA, not applicable; SARS-CoV-2, severe acute respiratory syndrome coronavirus 2.

^a^ Adjusted for age and sex.

^b^ For anonymity, hospitals are identified by number.

## Discussion

As COVID-19 pandemic reopening strategies are contemplated and enacted, understanding asymptomatic SARS-CoV-2 infection among HCWs is critical.^3,4^ We report a 4.8% difference between COVID-19–facing (5.4%) and non–COVID-19–facing (0.6%) HCWs, potentially indicating transmission from patients or coworkers.^5,6^ All nonclinical HCWs and community residents had RT-PCR test results negative for SARS-CoV-2. Nonclinical HCWs worked in buildings with separate entrances and heating, ventilation, and air conditioning systems, with lower population density due to remote working policies. Our comparison across job categories of COVID-19–facing HCWs did not yield significant differences between presumably high and low exposures, supporting the need for uniform infection control practices within patient care units.

Our findings are limited by convenience sampling from a single health care system and a small homogenous sample of community residents. However, higher infection rates among COVID-19–facing clinical HCWs and interhospital differences highlight the need for surveillance, isolation, and consistent infection control throughout an organization. Ongoing HCW surveillance is imperative to restore clinical operations.

## References

[1] Gandhi M, Yokoe DS, Havlir DV. Asymptomatic transmission, the Achilles’ heel of current strategies to control COVID-19. N Engl J Med. 2020;382(22):2158-2160. doi:10.1056/NEJMe2009758 32329972 PMC7200054

[2] Kimball A, Hatfield KM, Arons M, ; Public Health—Seattle and King County; CDC COVID-19 Investigation Team. Asymptomatic and presymptomatic SARS-CoV-2 infections in residents of a long-term care skilled nursing facility—King County, Washington, March 2020. MMWR Morb Mortal Wkly Rep. 2020;69(13):377-381. doi:10.15585/mmwr.mm6913e1 32240128 PMC7119514

[3] Day M. COVID-19: four fifths of cases are asymptomatic, China figures indicate. BMJ. 2020;369:m1375. doi:10.1136/bmj.m1375%J 32241884

[4] Guan WJ, Ni ZY, Hu Y, ; China Medical Treatment Expert Group for Covid-19. Clinical characteristics of coronavirus disease 2019 in China. N Engl J Med. 2020;382(18):1708-1720. doi:10.1056/NEJMoa2002032 32109013 PMC7092819

[5] World Health Organization. Coronavirus disease (COVID-19) situation report—84. Updated April 13, 2020. Accessed June 30, 2020. https://www.who.int/docs/default-source/coronaviruse/situation-reports/20200413-sitrep-84-covid-19.pdf?sfvrsn=44f511ab_2

[6] Wilder-Smith A, Teleman MD, Heng BH, Earnest A, Ling AE, Leo YS. Asymptomatic SARS coronavirus infection among healthcare workers, Singapore. Emerg Infect Dis. 2005;11(7):1142-1145. doi:10.3201/eid1107.041165 16022801 PMC3371799

